# β-lactamase (Bla) Reporter-based System to Study Flagellar Type 3 Secretion in *Salmonella*

**DOI:** 10.21769/BioProtoc.4696

**Published:** 2023-06-20

**Authors:** Fabienne F. V. Chevance, Kelly T. Hughes

**Affiliations:** School of Biological Sciences, University of Utah, Salt Lake City, United States of America

**Keywords:** β-lactamase, Secretion assays, *Salmonella*, Flagellar type-III secretion, Positive selection for secretion

## Abstract

Export of type 3 secretion (T3S) substrates is traditionally evaluated using trichloroacetic acid (TCA) precipitation of cultured cell supernatants followed by western blot analysis of the secreted substrates. In our lab, we have developed β-lactamase (Bla), lacking its Sec secretion signal, as a reporter for the export of flagellar proteins into the periplasm via the flagellar T3S system. Bla is normally exported into the periplasm through the SecYEG translocon. Bla must be secreted into the periplasm in order to fold into an active conformation, where it acts to cleave β-lactams (such as ampicillin) to confer ampicillin resistance (Ap^R^) to the cell. The use of Bla as a reporter for flagellar T3S allows the relative comparison of translocation efficiency of a particular fusion protein in different genetic backgrounds. In addition, it can also be used as a positive selection for secretion.

Graphical overview

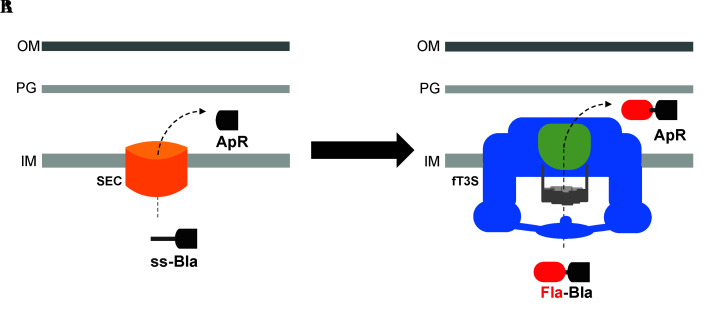

**Utilization of β-lactamase (Bla) lacking its Sec secretion signal and fused to flagellar proteins to assay the secretion of exported flagellar substrates, into the periplasm, through the flagellar T3S system.** A. Bla is normally transported into the periplasm space through the Sec secretion pathway, where it folds into an active conformation and allows resistance to ampicillin (Ap^R^). B. Bla, lacking its Sec secretion signal, is fused to flagellar proteins to assay the secretion of exported flagellar proteins into the periplasm through the flagellar T3S system.

## Background

Flagella are helical, corkscrew-like appendages that, depending on their clockwise or counterclockwise rotation, push or pull bacterial cells. They act like tiny propellers allowing bacteria to move through liquids or across hydrated surfaces. For the assembly of the bacterial flagellum, a flagellar type 3 secretion (T3S) system initially exports early component substrates that build the hook-basal body (HBB) structure, which is the main component making up the flagellar motor. Upon HBB completion, the flagellar T3S system undergoes a secretion substrate specificity switch, resulting in an export selectivity for late substrate proteins, which include flagellin that assembles into the long external filament that acts as the propeller of the flagellum.

The export of T3S substrates is traditionally evaluated using trichloroacetic acid (TCA) precipitation of the supernatant of cultured cells, followed by western blot analysis of the secreted substrates. This technique is very useful at assessing protein translocation but is limited to the study of substrates that are translocated outside the cells. We have developed a reporter assay using β-lactamase (Bla), lacking its Sec secretion signal, for the secretion of flagellar proteins into the periplasm through the flagellar T3S system. Bla is an enzyme that cleaves and inactivates β-lactam antibiotics, such as ampicillin (Ap), through hydrolysis of the peptide bond of the characteristic four-membered β-lactam ring. The inactivation of the antibiotic provides Ap-resistance (Ap^R^) to the bacterium; for that, Bla needs to be transported into the periplasm where it folds into an active conformation. Such translocation into the periplasm occurs through the Sec-secretion pathway. Secreted proteins through the Sec-dependent pathway are readily recognized by an N-terminal signal sequence, which is cleaved during the process of secretion into the periplasm to yield a mature protein. Flagellar proteins are exported through the T3S pathway. Fusing Bla without its N-terminal signal sequence to the C-terminal of flagellar proteins results in the transport of the Fla-Bla fusions into the periplasm, where Bla is active and confers Ap^R^ (Lee and Hughes, 2006; Hirano et al., 2009; Erhardt and Hughes, 2010; Singer et al., 2014; Hendriksen et al., 2021; Qu et al., 2022). In cells expressing intact flagellar structures, Fla-Bla fusions are secreted into the periplasm transiently after completion of the flagellar core T3S system until outer membrane penetration. Once the flagellar structure penetrates the outer membrane, the Fla-Bla fusions are secreted into the extracellular medium. Mutants defective in rod assembly continuously secrete Fla-Bla fusion into the periplasm, which results in higher levels of Ap^R^. By using assays to assess minimum inhibitory concentrations of ampicillin, we can estimate how much of the Fla-Bla fusion is exported and provide a quick estimate of secreted flagellar substrate levels. The use of Bla as a reporter for the flagellar T3S substrates also provides a positive selection for secretion. Using this technique, we were able, for example, to localize important sites of recognition in an early substrate’s secretion signal by the flagellar T3S apparatus located at the cytoplasmic base of flagellum (Qu et al., 2022). This system is not specific to *Salmonella*; Bla fusions to T3S proteins have been used in other Gram-negative pathogens, such as enteropathogenic and enterohemorrhagic *Escherichia coli* and *Yersinia enterocolitica* (Charpentier and Oswald, 2004; Diepold et al. 2015). In these systems, Bla fusions were used to measure the translocation of effector proteins in living host cells and in the extracellular medium, using a fluorescent β-lactamase substrate. Thus, fusion to Bla can be used to measure translocation of proteins either into the periplasm or the extracellular medium. We use secretion of Bla fusions in the periplasm because of the powerful selection of Ap^R^. Here, we describe in detail the protocol used to assay the minimum inhibitory concentration to ampicillin for the assessment of the translocation efficiency of protein fusions in the periplasm and for positive selections using this system.

## Materials and reagents

1. 96-well culture plates (Olympus plastic, catalog number: 25-104)
2. Test tubes (borosilicate glass 13 × 100 mm) (FisherBrand, catalog number: 14-961-27)
3. Petri dishes (9 cm diameter)
4. Microcentrifuge tubes (1.5 mL)
5. Conical centrifuge tubes (50 mL)
6. Millipore water, sterilized
7. Ampicillin sodium salt (Sigma-Aldrich, catalog number: A9518-100G)
8. Bacto peptone (Gibco, catalog number: 211677)
9. Difco bile salts No. 3 (BD, catalog number: BD 213020)
10. Bacto proteose peptone (Gibco, catalog number: 211684)
11. Agar, powdered (Apex, catalog number: 20-273)
12. Sodium chloride (Sigma-Aldrich, catalog number: S9888-1KG)
13. Yeast extract (Apex, catalog number: 20-254)
14. Tryptone, powdered (Apex, catalog number: 20-251)
15. Sodium phosphate dibasic heptahydrate (Na_2_HPO_4_·7H_2_O) (Fisher, catalog number: S373-500)
16. Potassium phosphate monobasic (KH_2_PO_4_) (Fisher, catalog number: P285-3)
17. Aluminum foil
18. Strains to be tested and containing fusions to β-lactamase (see Notes)
19. Lysogeny Broth-Lennox (LB) media (see Recipes)
20. PPBS plates (see Recipes)
21. 10× phosphate buffer (see Recipes)
22. Buffered saline (see Recipes)
23. Ampicillin solution stocks (see Recipes)
24. Lysogeny Broth-Lennox (LB) plates (see Recipes)

*Notes:*

- *We routinely use* Salmonella typhimurium *LT2, strain TH437 (S. typhimurium LT2 wild type) as a negative control (MIC 1.5) and strain TH15737 (flgM-bla ΔflgHI fljBenxvh2 Δflk) as a positive control (MIC 100). These strains are available upon request.*
- *We insert the Bla fusion without its signal sequence (amino acids 24–286 of Bla) just before the stop codon of the gene of interest, in the chromosome, using the lambda red recombination technique. We routinely do not add linkers, but we see no reason they could not be added. Confirm that the DNA sequence of any construct made is correct and that the protein expression is unaffected by the fusion before conducting assays.*

## Equipment

1. Conventional autoclave
-20 °C freezer
3. Vortex mixer
4. Temperature-controlled environmental shaker (set at 37 °C)
5. Temperature-controlled shaking water bath (set at 37 °C)
6. Standard micropipettes as well as matching tips
7. 2,000 μL electronic pipette repeater (Rainin EDP3-Plus LTS 0.2–2 mL)
8. 20 μL electronic pipette repeater (Rainin EDP3-Plus LTS 2–20 μL)
9. 25 mL glass pipettes, sterilized
10. 10 mL glass pipettes, sterilized
*Note: Multichannel pipettes can be used instead of pipette repeaters.*

## Procedure

**Secretion assays in liquid media using 96-well plates**

- **Prepare the cells**
  1. Inoculate three independent single colonies for each bacterial strain to be tested into 1 mL of LB media (see Recipes) supplemented with any required supplements. Make sure to include a strain that does not express β-lactamase fusion as a negative control.
  2. Grow the cells, under aeration, at 37 °C overnight.
  3. Dilute the cells 200-fold by pipetting 5 μL of the overnight cell culture into 995 μL of buffered saline (see Recipes).
- **Prepare the ampicillin solutions**
  1. Prepare a stock of 100 mg/mL ampicillin (Ap stock; see Recipes). Make 500 μL aliquots in microcentrifuge tubes and store at -20 °C until use. Prepare fresh every three months.
  2. Transfer 50 mL of LB media into a 50 mL conical centrifuge tube. Discard 400 μL of media using a pipette before adding 400 μL of the Ap stock in order to prepare the 800 μg/mL first solution. Mix well by vortexing. Label this tube number 1.
  3. Label 10 additional 50 mL tubes 2–11. Add 25 mL of LB media to each of them.
  4. Make the serial dilution by transferring 25 mL of Ap 800 μL/mL of conical centrifuge Tube 1 to conical centrifuge tube 2. Close and mix well, then perform the new transfer from tube 2 to 3, etc., until tube 10. Tube 11 contains only LB media as a control.
    At the end, we get the following serial dilutions:
    **Table:** 

1	800 μg/mL
2	400 μg/mL
3	200 μg/mL
4	100 μg/mL
5	50 μg/mL
6	25 μg/mL
7	12.5 μg/mL
8	6.25 μg/mL
9	3.125 μg/mL
10	1.565 μg/mL
11	0 μg/mL
*Notes:*
    - *This is just an example of a typical range used for one of our Bla fusion expression strains. A different range or more specific dilutions of ampicillin solutions can also be prepared, if necessary, in order to obtain a different range of concentrations.*
    - *Smaller volumes can be used to prepare the solutions; however, using larger volumes gives better reproducibility.*
- **Load the ampicillin solutions and cells on the 96-well plate**
  1. Calculate the number of plates needed, depending on the number of samples. Make sure to include control samples, such as buffered saline–only, to ensure all solutions are sterile. Place the plates such that they are facing 12 by 8, as illustrated in Figure 1.
    **Figure 1.**
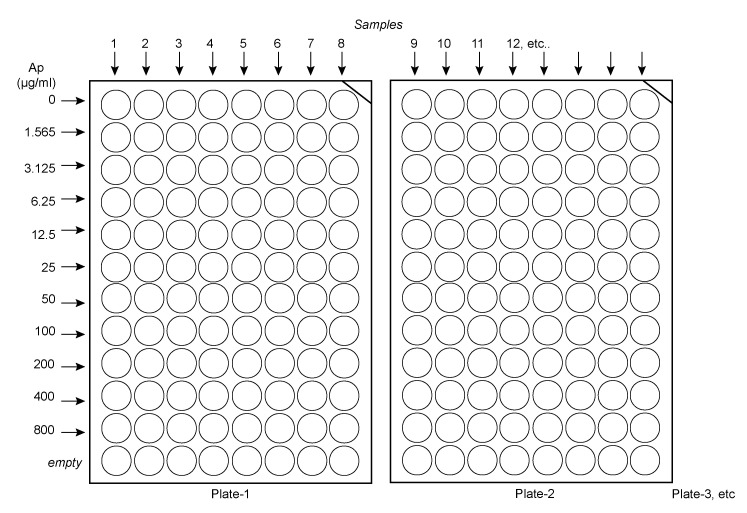
Suggested layout to load ampicillin solutions and samples in the plates
  2. Number each plate and write sample numbers on the plates.
  3. Using a 2,000 μL electronic pipette repeater, transfer 198 μL of each of the ampicillin solutions across the plates. Load solution 11 (no ampicillin) first, then solution 10 (1.565 μg/mL), etc., until the higher concentration solution.
  4. Once all plates are loaded with the Ap solutions, use a 20 μL electronic pipette repeater to load 2 μL of the 200-fold diluted cells to each well, starting from the least concentrated Ap solution down until the most concentrated solution.
  5. Place each plate on top of each other with their cap and tape them together and to the shaker plate in the incubator.
  6. Incubate at 37 °C and 180 rpm for 18 h.
*Notes:*
  - *Ampicillin is sensitive to light; keep the antibiotic in the dark throughout all steps. Use aluminum foil if necessary to protect the plates from the light.*
  - *While we routinely use ampicillin in our studies, carbenicillin might be a better option because of its increased stability.*

## Data analysis

1. Carefully remove the plates from the incubator and lay them in front of you with the lowest ampicillin concentration at the top.
2. Note in which wells bacteria have grown or not. The minimum inhibitory concentration (MIC) is defined as the first ampicillin concentration for which the cells are NOT growing. Figure 2 shows an example of typical results obtained. In this example, seven different samples were loaded and grown for 18 h at 37 °C. Sample 8 was buffered saline as a control. It is easy to see where samples are growing or not. Thus, in this example, sample 1 gives a MIC of 25; samples 2 and 3 give a MIC of 6.25; samples 4, 5, and 7 give a MIC of 50, and sample 6 gives a MIC of 12.5 μg/mL.
3. Record the results.
  **Figure 2.**
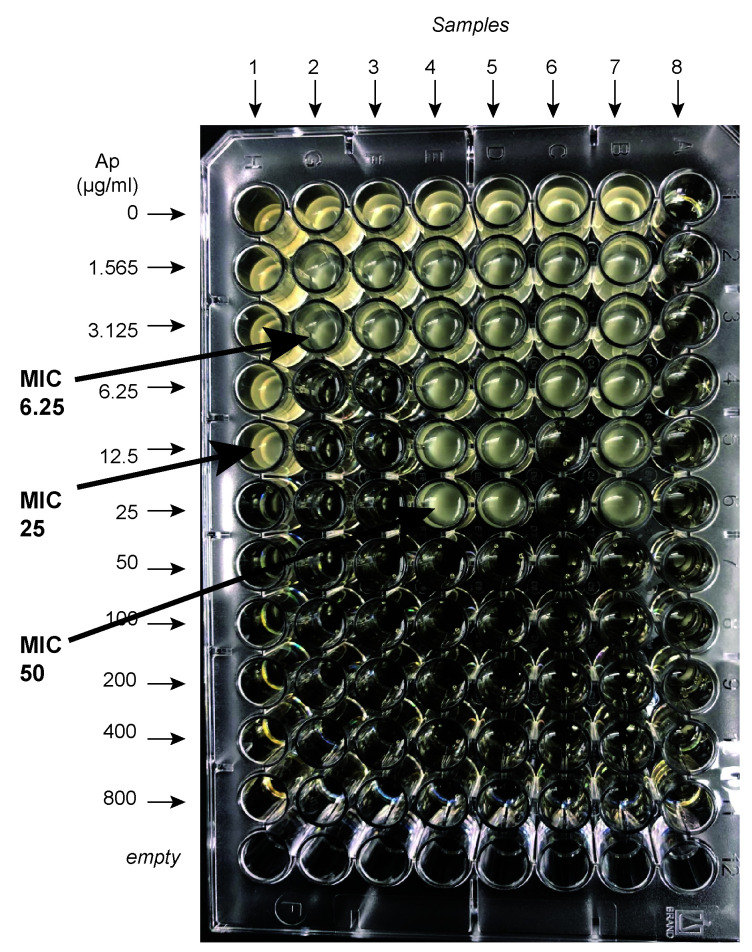
An example of a 96-well secretion assay. Seven different samples were loaded on this plate and grown for 18 h at 37 °C. Buffered saline was loaded on column 8. The minimum inhibitory concentration (MIC) is defined as the first ampicillin concentration for which the cells are NOT growing. Thus sample 1 gives a MIC of 25; samples 2 and 3 give a MIC of 6.25; samples 4, 5, and 7 give a MIC of 50, and sample 6 gives a MIC of 12.5 μg/mL.

*Notes:*

- *It is a good idea to conduct replicate analysis on different days and to include some internal controls (positive and negative) in each assay.*
- *We avoid the use of plasmid expression system for this assay in order to prevent artifacts associated with varying plasmid copy numbers in different cells. Chromosomally expressed Bla fusions are effective and give consistent, reproducible data.*
- *For the most part, three independent replicates give consistent MIC values (same value). For some genetic backgrounds, however, there might be inconsistencies in the MIC values obtained. In this case, repeat the assay with nine independent biological replicates for that particular genetic background, and take the most common value for MIC (found at least five times). If the results are still inconsistent, use solutions with a different range of ampicillin concentrations.*

**Secretion assays using solid medium containing bile salts**

We found that the utilization of bile salts helps to provide a more stringent screen or selection (Hirano et al., 2009) and can also be used for Ap^R^ assays on solid medium (Qu et al., 2022). The protocol we developed for such assay is as follows:

1. Inoculate three independent single colonies for each bacterial strain to be tested into 1 mL of LB media supplemented with any required supplements.
2. Grow the cells, under aeration, at 37 °C until the culture reaches approximately 2 × 10^9^ cells/mL (overnight culture).
3. Transfer 20 μL of the overnight culture into 2 mL of fresh LB media (100-fold dilution) and any required supplements.
4. Grow the freshly diluted cells for 90 min at 37 °C with aeration.
5. Dilute cells 1,000-fold into buffered saline, by pipetting 1 μL of the freshly grown cells into 1,000 μL of buffered saline.
6. Spot 4 μL of the 1,000-fold diluted onto PPBS plates (see Recipes) containing varying concentrations of Ap.
7. Incubate overnight at 37 °C.
8. Report the presence or absence of growth on all the PPBS-Ap plates with varying Ap levels. An example of a MIC assay using PPBS-Ap plates is shown in Figure 3.
  **Figure 3.**
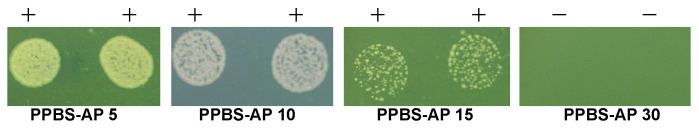
An example of secretion assay using solid medium containing bile salts
*Note: The resistance to ampicillin on PPBS-Ap plate is stronger than in Ap liquid media. The dilutions of cells that we used for the solid assay match the resistance obtained by simple streaking our mutants on PPBS plates. The advantage of using solid medium containing bile salts (PPBS) is that the selection is tight and shows only the strongest ampicillin resistant mutants. In Qu et al. (2022), we used both solid and liquid secretion assays. Using PPBS solid medium, we distinguished alleles with significant secretion of the Bla fusion. Using liquid secretion assays without bile salts, we could detect low levels of secretion of fusions with amino acid substitutions that were not observed to be secreted on the more stringent bile salts–containing solid medium.*

**PPBS-Ap plates used for selections**

We use Bla as a reporter for flagellar T3S, not only for the quantification of secreted flagellar protein levels, but also as a positive selection for secretion. We found that the key for Ap^R^ selections is to add bile salts to the plates, which helped to provide a stringent screen (Hirano et al., 2009). A typical protocol for selection on PPBS-Ap plates is described below:

1. Inoculate 10 independent single colonies, from a strain containing Bla, into 1 mL of LB media supplemented with any required supplements. Also, start a strain that does not express Bla (no-Bla) as a control.
2. Grow overnight cultures of the 10 independent colonies and the no-Bla control strain at 37 °C with aeration.
3. Plate 100 μL of each independent culture and the no-Bla control strain onto the appropriate PPBS-Ap plate. (Choose a 2-fold higher Ap concentration; for example, if the strain grows on PPBS-Ap5 plates but not on PPBS-Ap10, use PPBS-Ap10 for the selection.)
4. Next day, pick four Ap^R^ colonies from each independent selection and purify by streaking twice onto non-selective media (LB plates; see Recipes).
5. Recheck each individual colony for Ap^R^ and keep one colony from each independent selection.
6. Map the mutations or send to genome sequencing in order to identify the mutation responsible for the Ap^R^ phenotype.

## Recipes

1. **Ampicillin solution stocks**
  - For liquid assays:
    Prepare 10 mL stock solution of 100 mg/mL of ampicillin in water.
    Filter sterilize.
    Aliquot in 500 μL microcentrifuge tubes and keep at -20 °C until use.
    Use one single aliquot for each assay, to avoid freeze thawing.
  - For PPBS-Ap plates:
    Prepare 100 mL stock solutions of 6 or 20 mg/mL of ampicillin in 50% ethanol/50% water, filter sterilize, and keep at -20 °C in the dark
*Notes:*
*i. Ampicillin is sensitive to light; keep the antibiotic in the dark throughout all steps.*
*ii. Prepare fresh stock solutions of sodium ampicillin every three months.*
2. **Proteose peptone bile salt (PPBS) plates (1 L)**
  Prepare Flasks A and B as follows:
  **Table:** 

Flask A	Flask B
17 g of Bacto peptone 3 g of Bacto proteose peptone 10 g of NaCl 1.5 g of Difco bile salt #3 500 mL of distilled water	12 g of agar 500 mL of distilled water
  Place media flasks in an autoclave-safe bin containing a small amount of water and autoclave for 30 min. Add the desired concentration of ampicillin solution to flask A as soon as the flasks can be hand touched (approximately 55 °C). Mix Flask A and B by pouring contents of flask A into B and then back and forth two more times, to ensure the liquid is well mixed. Pour plates. Protect plates from light. Place plates in plastic bags after two days of drying at room temperature. Place in a cardboard box to protect from light and store at 4 °C. Plates can be used for at least one month.
3. **Lysogeny LB plates (1 L)**
  Prepare flasks A and B as follows:
  **Table:** 

Flask A	Flask B
10 g of tryptone 5 g of yeast extract 5 g of sodium chloride 12 g of agar 500 mL of distilled water	500 mL of distilled water
  Place media flasks in an autoclave-safe bin containing a small amount of water and autoclave for 30 min. Once the flasks can be hand touched (approximately 55 °C), mix Flask A and B by pouring contents of flask B into flask A and then back and forth two more times to ensure the liquid is well mixed. Pour plates. Place plates in plastic bags after two days of drying at room temperature. Store at 4 °C. Plates can be used for at least three months.
4. **Lysogeny LB media (1 L)**
  Weigh the following ingredients and dissolve into 1,000 mL of distilled water:
  10 g of tryptone
  5 g of yeast extract
  5 g of sodium chloride
  Aliquot into 200 mL bottles and autoclave for 30 min. Store autoclaved bottles at room temperature until use.
5. **10× phosphate buffer (1 L)**
  Weigh the following ingredients and dissolve into 500 mL of distilled water:
  110 g of Na_2_HPO_4_·7H_2_O
  30 g of KH_2_PO_4_
  Adjust pH to 7 and add water up to 1 L. Sterilize by autoclaving.
6. **Buffered saline (1 L)**
  Weigh the following ingredients and dissolve into 1,000 mL of distilled water:
  8.5 g of sodium chloride
  100 mL of 10× phosphate buffer
  Aliquot into 200 mL bottles and autoclave for 30 min. Store autoclaved bottles at room temperature until use.
